# Tau modulation through AAV9 therapy augments Akt/Erk survival signalling in glaucoma mitigating the retinal degenerative phenotype

**DOI:** 10.1186/s40478-024-01804-0

**Published:** 2024-06-07

**Authors:** Kanishka Pushpitha Maha Thananthirige, Nitin Chitranshi, Devaraj Basavarajappa, Rashi Rajput, Mojdeh Abbasi, Viswanthram Palanivel, Veer Bala Gupta, Joao A. Paulo, Maya Koronyo-Hamaoui, Mehdi Mirzaei, Stuart L. Graham, Vivek Gupta

**Affiliations:** 1 Faculty of Medicine, Health and Human Sciences, Macquarie Medical School, Macquarie University, Sydney, NSW 2109 Australia; 2 Division of Ophthalmology, Department of Biomedical and Clinical Sciences, Linköping University, 58183 Linköping, Sweden; 3 School of Medicine, Deakin University, Melbourne, VIC 3220 Australia; 4 Department of Cell Biology, Harvard Medical School, Boston, MA USA; 5 Department of Neurosurgery, Cedars-Sinai Medical Center, Maxine Dunitz Neurosurgical Research Institute, 127 S. San Vicente Blvd., Los Angeles, CA 90048 USA; 6 Division of Applied Cell Biology and Physiology, Departments of Neurology and Biomedical Sciences, Cedars-Sinai Medical Center, 127 S. San Vicente Blvd., Los Angeles, CA USA

**Keywords:** Glaucoma, Tau, Retinal ganglion cells, Microtubules, Neurodegeneration, Intraocular pressure, Optic nerve

## Abstract

The microtubule-associated protein Tau is a key player in various neurodegenerative conditions, including Alzheimer's disease (AD) and Tauopathies, where its hyperphosphorylation disrupts neuronal microtubular lattice stability. Glaucoma, a neurodegenerative disorder affecting the retina, leads to irreversible vision loss by damaging retinal ganglion cells and the optic nerve, often associated with increased intraocular pressure. Prior studies have indicated Tau expression and phosphorylation alterations in the retina in both AD and glaucoma, yet the causative or downstream nature of Tau protein changes in these pathologies remains unclear. This study investigates the impact of Tau protein modulation on retinal neurons under normal and experimental glaucoma conditions. Employing AAV9-mediated gene therapy for Tau overexpression and knockdown, both manipulations were found to adversely affect retinal structural and functional measures as well as neuroprotective Akt/Erk survival signalling in healthy conditions. In the experimental glaucoma model, Tau overexpression intensified inner retinal degeneration, while Tau silencing provided significant protection against these degenerative changes. These findings underscore the critical role of endogenous Tau protein levels in preserving retinal integrity and emphasize the therapeutic potential of targeting Tau in glaucoma pathology.

**Supplementary Information:**

The online version contains supplementary material available at 10.1186/s40478-024-01804-0.

## Introduction

Glaucoma, a chronic neurodegenerative disorder, induces irreversible vision loss by progressively damaging retinal ganglion cells (RGCs) and the optic nerve, often associated with heightened intraocular pressure (IOP) [14]. Therapeutic lowering of IOP is beneficial but the underlying pathology has been shown to progress in several cases. This has necessitated a comprehensive understanding of the molecular mechanisms driving glaucoma pathology [36]. Cumulative evidence has shown Microtubule-associated protein Tau (MAPT) hyperphosphorylation in the retina in glaucoma conditions [32]. Additionally, Tau aggregates have been observed in the retinas of mice carrying the human mutated Tau 301S [35].

Beyond glaucoma, the Tau protein has been shown to play a pivotal pathognomonic role in other neurodegenerative conditions such as Alzheimer’s disease (AD), Parkinson’s disease (PD), Frontotemporal dementia (FTD), Pick Disease (PiD), corticobasal degeneration (CBD), and progressive supranuclear palsy (PSP) [23]. Abundantly expressed in neuronal cells in the brain and retina, Tau is crucial for stabilizing the microtubular network, which regulates intracellular transport and preserves cellular morphology [41]. The hyper-phosphorylated forms of Tau have been reported to induce protein aggregation leading to neuropil thread and neurofibrillary tangle (NFT) formation, which are cytotoxic and have been implicated in mediating neuronal loss and associated cognitive deficits [41]. Tau protein alterations have been reported in cerebrospinal fluid (CSF) and plasma in AD and other Tauopathies [30].

Aberrant Tau phosphorylation disrupts cellular signalling networks, diminishing Tau's affinity for axonal microtubules, thereby destabilizing them and adversely affecting normal axonal transport [42]. Microtubular networks define the cellular morphology, regulate structural elasticity and integrity, and impart stability that is critical for neuronal function and synaptic signalling [17]. In physiological conditions, Tau stabilizes globular tubulin subunits, crucial building blocks for microtubules [21]. Stable forms of tubulin are more acetylated and significantly hyper phosphorylated forms of Tau undergo detachment from the acetylated microtubules due to electrostatic repulsions [2]. A chronic microtubular impairment followed by the axonal destabilization have been observed in AD [13].

Further, Tau hyperphosphorylation and associated molecular changes, such as cytoskeletal vulnerability promote unfolded protein response (UPR) in cells leading to endoplasmic reticulum (ER) stress pathway activation [6]. Under cellular stress conditions, there is an enhanced binding of BiP with ER lumen proteins such as IRE-1α and PERK [22]. BiP is involved in mediating normal protein folding in the ER lumen and its dysfunction causes ER stress sensor activation [22]. CHOP is another key ER stress marker that interacts with BCL-2, an anti-apoptotic protein and promotes caspase–3 activation [20]. CHOP induction has previously been shown to occur in a p-PERK mediated pathway, and under the stress conditions BiP, an early stress protein gets detached from the PERK, leading to enhanced PERK phosphorylation [25]. Moreover, pPERK has also been shown to interact with p-elf2α and induce CHOP expression, which is expressed in the late ER stress response and promotes apoptosis [45]. Elevated levels of ER stress markers have been reported in the glaucomatous retina [7, 11]. This study investigates the interplay between Tau protein and ER stress response proteins in experimental glaucoma conditions.

Furthermore, the Ser/Thr specific protein kinase B or Akt is integral in regulating glucose metabolism, cell migration, and cellular proliferation [9]. Akt-mediated GSK3 deactivation through phosphorylation of GSK3α at Ser 21 and GSK3β at Ser 9, has been implicated in the regulation of Tau protein phosphorylation [9]. Thus, Akt activation or its downregulation may affect the specific phosphorylation profile of Tau protein [9]. This alteration in Tau phosphorylation mediated by GSK3β has been shown in vitro as well as in human and animal brain tissues [12, 49]. GSK3β can phosphorylate multiple residues in Tau and reduce its ability to interact with the microtubules leading to microtubular network destabilization [33]. On the other hand, GSK3-mediated phosphorylation has been shown to augment the phospho- Tau interactions with chaperone protein prolyl isomerase 1 (PIN–1) [28]. Tau protein aggregation has been suggested to promote PERK-mediated GSK3β activation [1], triggering pro-apoptotic pathways leading to axonal degeneration and cell death. Additionally, Tau serves as a substrate for caspase-3 which induces Tau truncation at Asp421 site and lead to protein aggregation and neuronal apoptosis in AD [34].

This study aimed to assess the impact of modulating Tau expression in the mouse retina under normal and experimental glaucoma conditions. A comprehensive evaluation of retinal laminar structural and functional changes was conducted to determine the effect of Tau expression modulation. Furthermore, investigations into the impact of altered Tau levels on ER stress markers, Akt/Erk cellular survival signalling pathways, and synaptic markers provided crucial insights into disease mechanisms.

## Material and methods

### Animals

Animal studies were conducted in adherence to the guidelines outlined by the Association for Research in Vision and Ophthalmology (ARVO) for the use of animals in ophthalmic and vision research, as well as in accordance with the Australian code of practice for the care and use of animals for scientific purposes. Approval for all experimental protocols in this study (reference number 2019/009) was obtained from the animal ethics committee of Macquarie University, NSW, Australia. Male C57BL/6 J mice, aged four to six weeks, were procured from the Animal Research Centre, Perth, WA, Australia, and were housed in a controlled room temperature environment at 21 °C with a fixed 12 h light/dark cycle. The animals were divided into five groups, each consisting of 10 mice: (1) the control group, (2) AAV9-GFP control (3) AAV9-mTau overexpression (OV) (4) AAV9-Tau scramble control group and (5) AAV-mTau knockdown (KD) group. One eye of each mouse received ocular injections of microbeads to induce high-IOP-glaucoma, while the other eye was injected with an equivalent volume of PBS, serving as the control group. The "n" value shown in each figure corresponds to the number of tissue samples collected from different animals within the same group. Anaesthesia was induced through intraperitoneal injection of a combination of 50 mg/kg ketamine and 0.5 mg/kg medetomidine, with subsequent reversal of anaesthesia achieved by a subcutaneous injection of 0.75 mg/kg atipamezole. Pain relief, when necessary, was administered using carprofen (2.5 mg/kg, i.p). Cephazolin (Ciloxan; Alcon Laboratories, Australia) and maxidex eye drops (Alcon Laboratories) were applied to both eyes, while lubricant ointment (Lacri-lube; Allergan, NSW, Australia) was utilized to prevent corneal drying until the animals had fully recovered.

### Antibodies

Anti—Tau (4019), anti—pTau (Ser 404) (20,194), anti—BiP (3177), anti—pAKT (4060), anti—AKT (4685), anti—pERK (4370), anti—ERK (4695), anti—pGSK3β (9323), anti—GSK3β (9315), anti—Acetylated Tubulin (39,715) and cleaved Caspase-3 (Asp175) (9661) antibodies were obtained from the cell signalling, MA, USA. Anti—GADD153 (SC7351) and anti—pPERK (SC3257) were obtained from Santa Cruz Biotechnology, CA, USA. Anti—Tyrosine Tubulin (T9028) and anti—pTau (Ser 199/Ser202) (44768G) and anti—PSD95 (516,900) antibodies were obtained from Thermo Fisher Scientific, MA, USA. Anti—total tubulin (ab7751), anti—actin (ab6276), anti—synaptophysin (ab32127) anti—NeuN (ab104225 & ab104224) and anti—GFP (ab290 & ab1218) antibodies were obtained from the Abcam, VIC, Australia. Alexa—Fluor 488 conjugated affini pure donkey anti mouse IgG (715-545-150), Cy 3—conjugated affini pure donkey anti rabbit IgG (711-165-152) and Fluorescein (FITC) conjugated affini pure donkey anti goat IgG (705-095-147) were purchased from the Jackson Immuno Research Laboratories, Inc., PA, USA. Rabbit IgG horseradish peroxidase—conjugated antibody (HAF 008), Mouse IgG horseradish peroxidase-conjugated antibody (HAF 018), and goat IgG horseradish peroxidase-conjugated antibody (HAF 019) were acquired from the R&D Systems, Inc., MN, USA.

### Electrophysiological studies

The animals underwent overnight dark adaptation in a designated dark room before being anesthetized. Topical anaesthesia, comprising 1% alcaine and 1% tropicamide, was applied to the cornea to facilitate pupil dilation. To maintain the animals' body heat during the procedure, they were positioned on a heated sliding stage set at 37 °C. Subcutaneously placed ground and reference electrodes were situated in the tail and forehead, respectively. Custom-made gold ring electrodes, designed to minimize background noise, were carefully positioned on each eye, making contact with the cornea. To ensure a close connection between the electrodes and corneal tissue, a drop of methylcellulose was applied. Retinal electroretinograms (ERGs) were recorded using a Ganzfeld ERG machine with a flash intensity of 3 log cd·s/m^2^ (Phoenix-Micron, NY, USA). In ERG recording, the amplitude of the a-wave was measured from the origin to the lowest point in the trough, while b-wave amplitudes were measured from the trough to the crest of the b-wave. For the positive scotopic threshold response (pSTR), 0.5 Hz flash intensities of −4.3 log cd·s/m^2^ were delivered 30 times. In all positive scotopic threshold responses, amplitudes were measured from the baseline to the positive peak observed around 120 ms [8].

### Experimental glaucoma model

The animals were anesthetized using isoflurane (2%), and 1% tropicamide applied to dilate the pupils. One eye of each animal was injected with 2 µL microbead solution (3.6 × 10^6^ microbeads/mL) (weekly, 8 weeks) (Fluospheres, 10 μm, Molecular Probes) into the anterior chamber and the contralateral eye used as control. Hamilton syringes were used with 33-gauge disposable needles (TSK Laboratory, Tochigi, Japan). To avoid contact of the needle with the iris or lens, all the procedures were performed under the operating microscope (OPMI Vario S88, Carl Zeiss, Oberkochen, Germany). Once the needle was inserted beneath the corneal surface, microbead solution was injected. IOP measurements were taken by a handheld iCare tonometer (TonoLab, Icare, Finland) as described previously. Animals were kept on a warming pad until they recovered from anaesthesia [8, 16].

### Adeno-associated viral constructs

For the over expression of the Tau, murine mutant Tau (P232S) cDNA (NCBI ref: BC014748) under the transcriptional control of the cytomegalovirus chicken β-actin (CAG2) hybrid promotor was inserted into the adeno-associated virus serotype 9 (AAV9) vector with the enhanced green fluorescence protein reporter (eGFP) and 2A linker in between GFP and the mTau sequence (AAV9-CAG2-eGFP-2A-mTau (P232S)-WPRE or AAV9- Tau) (Vector Biolabs, USA). For Tau silencing, Tau shRNAmir was used to knock down the expression of the Tau (AAV9-CAG-eGFP-mTau-shRNAmir (sequence 5′-ACAGAGTCCAGTCGAAGATT-3′) or AAV9-Tau KD). AAV9—eGFP was used as the control for the AAV9-Tau and scramble (sequence 5′-CCACTACCGTTGTTATAGGTG-3′) as control for Tau knockdown construct. Animals were anesthetized by using isoflurane (2%) and tail gently dipped in warm water (45–50 °C) to dilate the veins. The viral vectors stocks were diluted with the PBS to obtain a final concentration of 1.0 × 10^10^ vg in a final 50 μl volume. The tail was cleaned using ethanol and 2% povidone—iodine solution. The 29G needle was inserted into the lateral tail vein at a 45° angle and viral solution injected [4].

### Tissue harvesting and processing

Animals were humanely euthanized through intraperitoneal injection of sodium pentobarbitone, and tissues were promptly collected. For Western blot (WB) analysis, tissues were gathered immediately post-euthanasia, collected retinal tissues snap frozen in liquid nitrogen, and preserved in a −80 °C freezer. Tissue samples were sonicated in lysis buffer (20 mM HEPES, pH 7.4, 1% Triton X-100, 2 mM EDTA) containing protease inhibitors (10 μg/ml aprotinin, 10 μM leupeptin, 1 mM phenylmethylsulfonyl fluoride) and phosphatase inhibitors (1 mM NaVO_3_, 100 mM NaF, 1 mM Na_2_MoO_4_, and 10 mM Na_2_P_2_O_7_) on ice. Following sonication, tissues were incubated for 30 min on ice and then centrifuged at 13,000 g for 10 min at 4 °C. The resulting supernatant was isolated and stored at −80 °C for subsequent analysis. For histological examination, animals underwent transcardial perfusion with 4% paraformaldehyde (PFA, Sigma). Harvested tissues were immersed in 4% PFA at 4 °C for 2 h. Tissues designated for paraffin-histological analysis were transitioned to 70% ethanol after three PBS washes for 5 min each, processed using an automatic tissue processor (ASP200S, Leica). Processed tissues were embedded in paraffin, and sections (5–8 µm) were obtained using a rotary microtome. Tissues intended for immunofluorescence analysis were immersed in a 30% sucrose solution in PBS. Incubation in 30% sucrose at 4 °C continued until complete sinking occurred. These tissues were embedded in optimal cutting temperature compound (OCT) cryostat embedding medium (Sakura Finetek), and sections of 8–10 µm thickness were prepared [4, 8].

### Histological analysis

For the hematoxylin and eosin (H&E) staining, paraffinized sections were used. 100% xylene was used to deparaffinize the sections following which they were rehydrated using graded ethanol solutions of 100, 95, and 50%, respectively. Slides were placed in hematoxylin solution (Sigma, USA) for 5 min, then rinsed with distilled water and then transferred to 0.1% Eosin Y (Sigma, USA) solution for 2–3 min. The slides were then transferred to 100% ethanol and xylene and mounted with a coverslip using a mounting medium as reported previously [4]. For immunostaining, tissues were subjected to sectioning using a cryostat. Sections were blocked with the blocking buffer for 1 h at room temperature. The sections were incubated with the primary antibody overnight at 4 °C. Slides were washed three times with 1x PBS, the secondary antibody diluted in antibody dilution buffer and incubated for 1 h in the dark at room temperature. This was followed by washing the slides thoroughly to remove any unbound antibodies and mounted using anti-fade mounting media with DAPI and stored at 4 °C in the dark. Zeiss fluorescence microscope (ZEISS Axio Imager Z2, Carl Zeiss, Oberkochen, Germany) was used for imaging, and images were quantified using Image J (NIH, USA) as reported previously [8, 11]. Cell density within the ganglion cell layer (GCL) of each eye was assessed by counting the cells within a 500 μm distance (ranging from 100 to 600 μm from the optic disc edge) across both the superior and inferior regions of the retina.

### Western blotting

Bicinchoninic acid assay (BCA) protein assay kit (Pierce, Rockford, USA) was used to estimate the protein quantity in the retinal lysates. The samples were loaded to NuPAGE 10% Bis–Tris gel (Invitrogen) using 1x MOPS SDS running buffer (Invitrogen). The separated proteins were transferred to the PVDF membrane (Invitrogen) using iBlot 2 gel transfer (Invitrogen). The membranes were blocked with the 5% skimmed milk in 1x TBST (20 mM Tris- HCl [pH 7.4], 100 mM NaCl, and 0.1% Tween 20) and incubated for 1 h at room temperature. The blots were washed the next day, incubated with horseradish peroxidase (HRP)-linked secondary antibodies (anti-rabbit 1:5000, and anti-mouse 1:5000, Jackson ImmunoResearch Labs), and incubated for 1 h at room temperature. Super Signal West Pico Chemiluminescent substrate (Pierce) was used to detect the bands in western blots using a luminescent image analyzer (ImageQuant LAS 4000, GE Healthcare). ImageJ software (NIH, USA) was used to quantify the band intensities within the linear detection range and data plotted [4, 8, 11].

#### Statistical analysis

GraphPad Prism software (version 6.0) (GraphPad Software, San Diego, CA) was used to analyse the data. All the data with the error bars are the mean ± SD and the number of samples (*n*) indicated in each figure represents the tissues from the different animals of the same group. The student t-test used for analysing unpaired two data sets. One-way ANOVA with Tukey’s multiple comparison test was done for analysing the multiple data sets. Results were significant if *p* < 0.05.

## Results

### AAV9 mediated Tau expression and phosphorylation changes in the retina

**Fig. 1:**
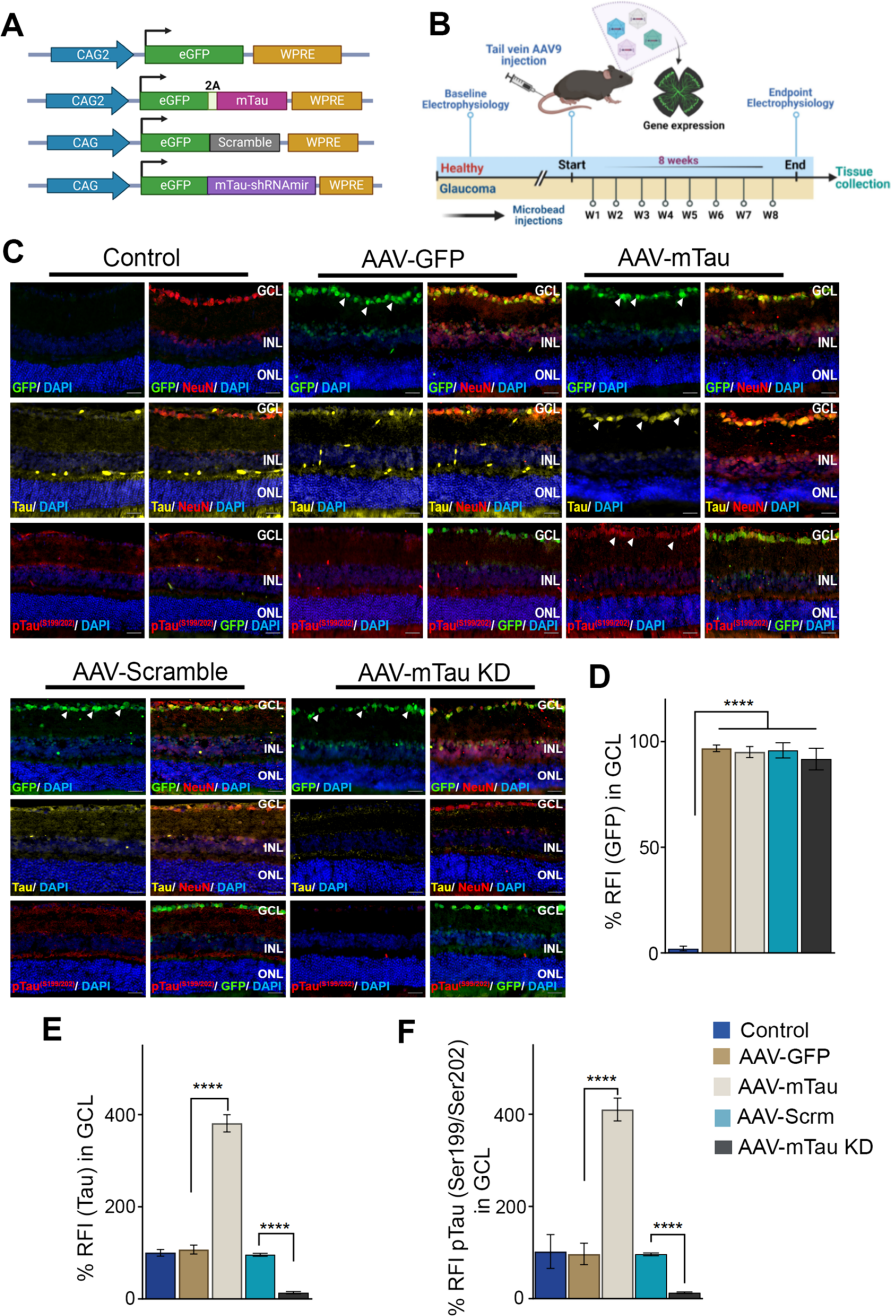
Modulation of Tau expression in C57BL/6 mice retinas **A** Scheme of AAV plasmid vector containing GFP, mTau, scrambled shRNA, and mTau-shRNAmir sequences. mTau (overexpression) and mTau-shRNAmir (knockdown, KD) sequences were cloned in AAV9 viral vector plasmid fused to the ampicillin-resistance gene. For mTau protein expression, a T2A self-cleaving peptide sequence was used. **B** Schematic depicting the experimental design and AAV administration experimental timeline. **C** Immunofluorescence images of retinal sections from control, AAV-GFP, AAV-mTau, AAV-Scramble and AAV-mTau KD groups showing GFP (green, FITC), tau (Green, Alexa Fluor 488), and ptau^(Ser199/Ser202)^ (Red, Cy3). NeuN (red-Cy3) and nucleus (blue, DAPI) (representative images, Scale bar = 50 μm, arrows indicate the changes in the expressions; Antibody concentrations: GFP (1:1000), tau (1:800), ptau^(Ser199/Ser202)^ (1:800), NeuN (1:1000)). **D** Quantification of GFP relative fluorescence intensity (RFI) percentage (****P < 0.0001, n = 5). **E** Quantification of Tau RFI percentage (****P < 0.0001, n = 5) **F** Quantification of pTau^(Ser199/Ser202)^ RFI percentage (****P < 0.0001, n = 5). Statistical significance was assessed by employing One-way ANOVA analysis with Tukey’s multiple comparison test

Mice were subjected to Tau overexpression (mTau) or Tau knockdown (KD) under the CAG2 hybrid promoter along with GFP as tracer using AAV9 constructs (Fig. 1A). The animals were monitored for 2 months after AAV administration, and retinal tissues examined to determine changes in Tau levels (Fig. 1B). Retinal sections from the control, Tau over expression (mTau) and GFP controls mice were stained against Tau, pTau (Ser^199^/Ser^202^) and GFP reactivity (Fig. 1C). Quantification revealed significantly increased levels of Tau immunoreactivity (2.73 ± 0.23 RFI fold change, *P* < 0.0001) and pTau (Ser199/Ser202) (3.12 ± 0.37 RFI fold change *P* < 0.0001) in the ganglion cell layer of Tau overexpression animals compared to the control GFP alone retinas (Fig. 1E, F). The pTau (Ser199/Ser202) reactivity was also observed to co-localise with the GFP staining in the GCL (Fig. 1C). A comparable level of GFP immunoreactivity were observed in the GFP alone and Tau-GFP expressing animals (*P* < 0.0001) (Fig. 1D). Further, Tau KD mice retinas were subjected to IF analysis to determine changes in Tau protein expression. A decreased Tau (*P* < 0.0001), pTau (Ser199/Ser202) (*P* < 0.0001) immunoreactivity was observed in the GCL of Tau KD subjected mice compared to the control retinas expressing scrambled sequence (Fig. 1E, F). A comparably increased levels of GFP immunoreactivity were evident in the scramble sequence and Tau KD subjected mice retinas (*P* < 0.0001) (Fig. 1D).

Changes in the Tau, GFP, pTau (Ser^199/202^) and pTau (Ser^404^) expression were further evaluated using western blotting analysis of the retinal lysates in both AAV-Tau overexpression and AAV-Tau KD experimental groups (Fig. S1A). The western blot intensities were quantified using β-actin as a loading control in each case and data plotted to determine changes in the protein expression levels. We observed significantly increased levels of Tau (6.54 ± 0.21-fold, *p* < 0.0001), pTauSer^199/^Ser^202^ (6.06 ± 0.15-fold, *P* < 0.0001) and pTau Ser^404^ (6.62 ± 0.12-fold, *P* < 0.0001) in AAV9-Tau over expression retinal lysates compared to GFP expressing control groups. A significant decrease in Tau (5.07 ± 0.04-fold, *P* < 0.001), pTau Ser^199^/Ser^202^ (8.81-fold ± 0.07 folds, *P* < 0.001) and pTau ^Ser404^ (5.02 ± 0.12-fold, *P* < 0.001) levels in AAV9-Tau KD animal retinal lysates was observed compared to scrambled sequence expressing control groups (Fig. S1B-D). A comparable GFP expression was evident in all AAV9 treatment-subjected retinal tissues from control and Tau expression modulated groups (Fig. S1E). Further, western blot results indicated no significant impact on the expression of APP in response to Tau modulation (Fig. S2A-B).

### Tau modulation induced functional and structural degenerative changes in the inner retina

**Fig. 2:**
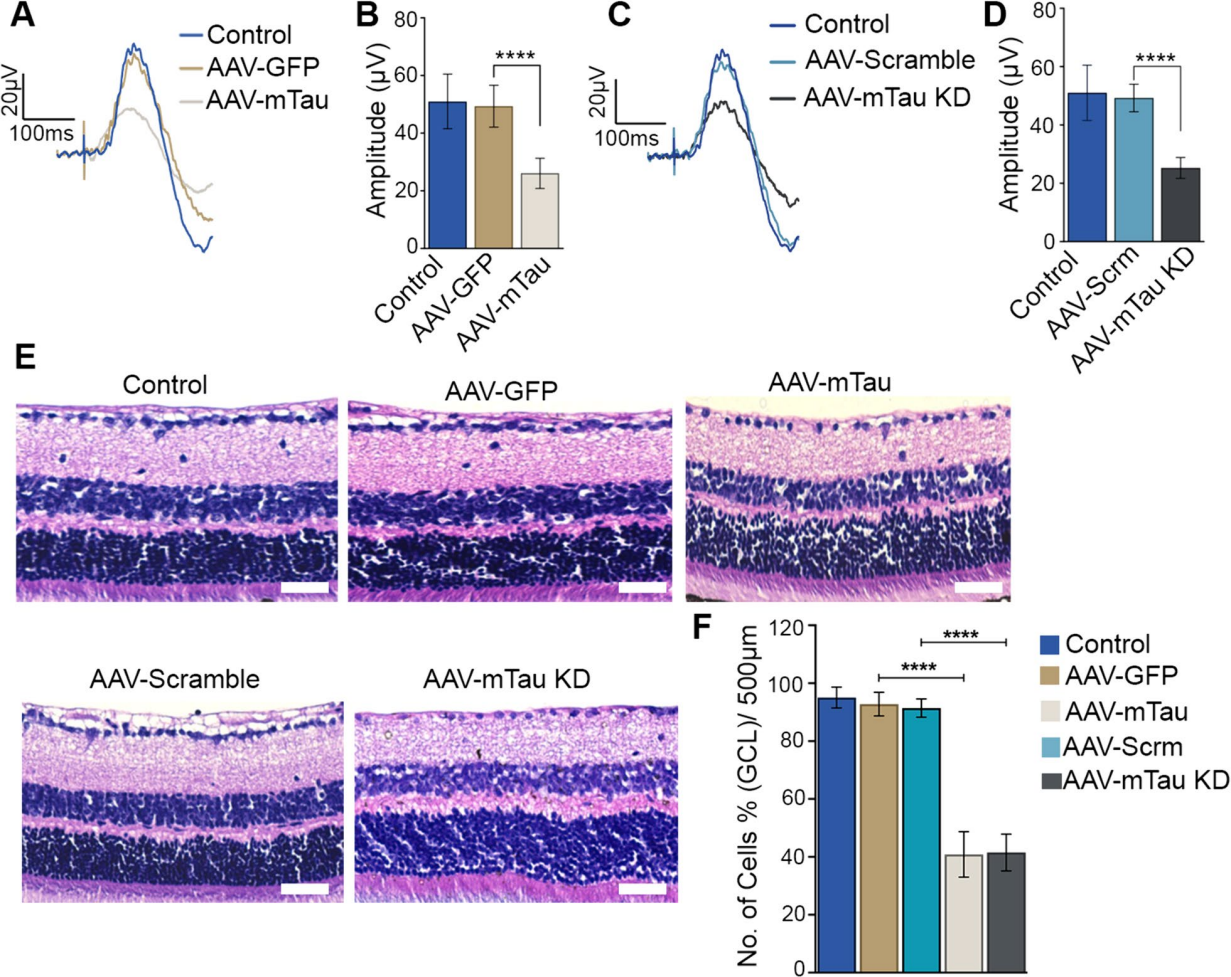
Electrophysiological and structural alterations in C57BL/6 mice retinas subjected to AAV-tau overexpression and AAV-tau knockdown under normal IOP conditions. **A** Positive scotopic threshold responses (pSTRs) of the control, AAV-GFP, and AAV-mTau injected mice. **B** Quantification of amplitudes indicates a significant decrease in pSTR amplitudes in AAV9-Tau overexpressing mice (n = 10, *P* < 0.0001 t-test). **C** pSTRs of the control, AAV-scrambled, and AAV-tau shRNA expressing mice and **D** their quantification indicates a significant decrease of pSTR amplitudes in AAV9-KD mice retinas (n = 10, ****P < 0.0001, t-test). No significant difference in pSTR amplitudes was observed between the control and AAV9-GFP or AAV9-scramble-shRNA sequence-expressing eyes. **E** H and E staining of retinal sections shows GCL cell density loss in Tau overexpression and Tau KD retinas compared to the controls, arrows indicate the changes in cell densities (representative images, Scale bar = 50 μm). **F** Quantification of cell density in the GCL from the retinal H and E-stained images (****P < 0.0001, One-way ANOVA analysis with Tukey’s multiple comparison test, n = 5 for each group)

The effects of Tau overexpression and Tau knockdown on the retinal functional changes in the mice retina were assessed using positive scotopic threshold response (pSTR) and whole retinal electroretinogram (ERG) measurements (Figs. 2A-D, S3). AAV9 expressing GFP alone was used a control for the Tau overexpression animals and AAV9 expressing scrambled sequence along with GFP was used as control for the Tau KD mice. The animals were monitored for 2 months subsequent to Tau protein modulation. A significant decline in pSTR amplitudes was observed for the Tau overexpression compared to the GFP alone expressing eyes (47.76 ± 5.25%, *P* < 0.0001) (Fig. 2B). Similar to that of the Tau overexpression model, a significant loss of pSTR amplitudes was also evident in Tau KD subjected model compared to the control AAV9 scramble shRNA expressing eyes (48.17 ± 3.57%, *P* < 0.0001) (Fig. 2D). The whole retinal full-field scotopic ERG analysis revealed no significant change in amplitudes, suggesting that the retinal functional changes were predominantly localised to the inner retina (Fig. S3A, B). Further, we sought to determine the impact of Tau protein modulation on the retinal laminar structure. H and E staining of the retinal sections revealed a significant decline in the ganglion cell layer (GCL) density in AAV9-Tau overexpression (51.95% ± 7.85%) and AAV9-Tau knockdown (49.92% ± 6.34%) retinas compared to the respective controls (*P* < 0.0001) (Fig. 2E, F). This experiment highlighted that an optimal Tau protein expression is essential to preserve the inner retinal function and structure.

### Tau overexpression exacerbated inner retinal degenerative changes induced by glaucoma

**Fig. 3:**
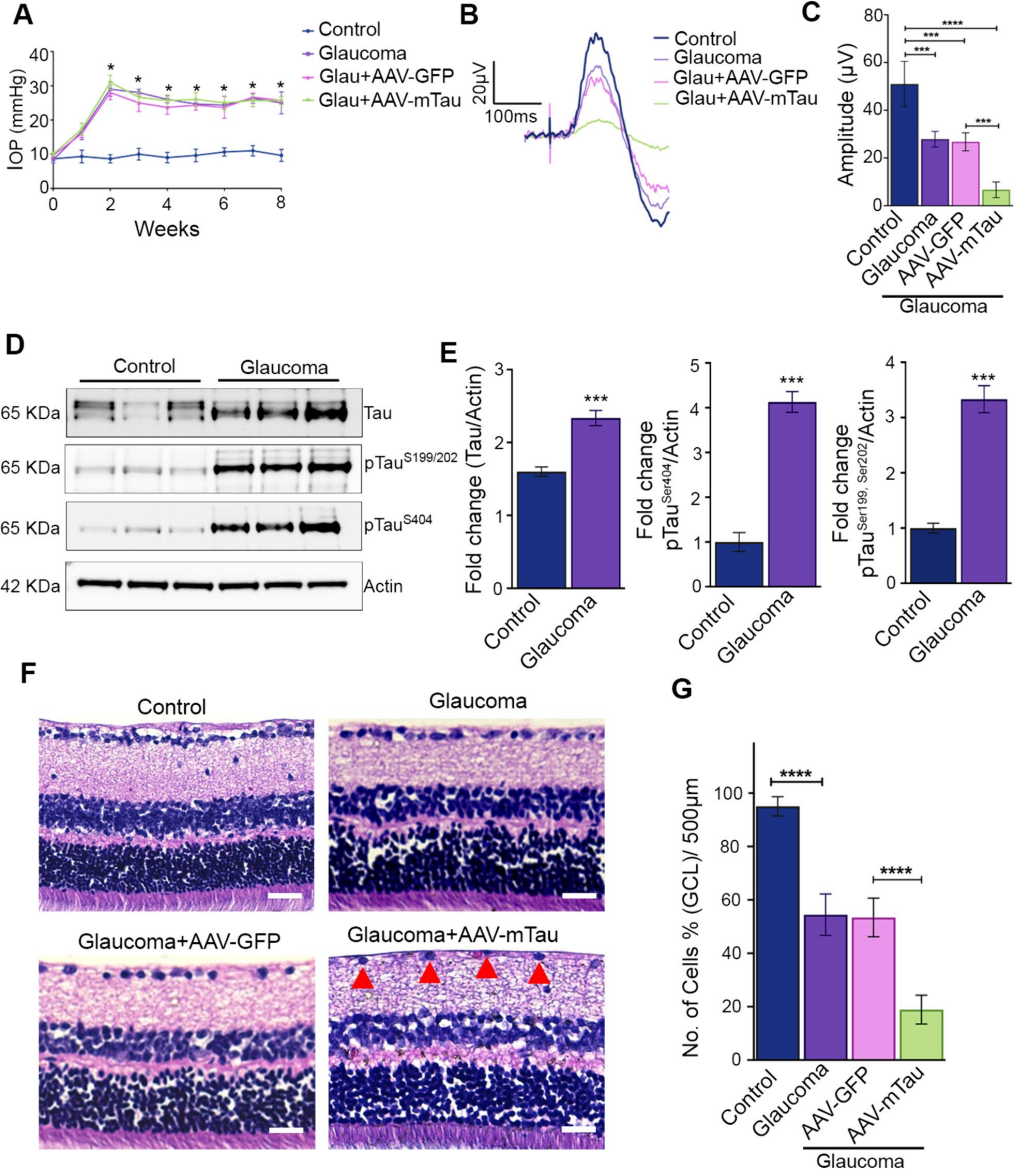
Tau overexpressing mice retinas shows exacerbated degenerative changes in the inner retina in high-IOP glaucoma condition. **A** Chronic elevation of IOP in C57BL/6 mice eyes was induced by intracameral microbead injections in control, AAV-GFP and AAV-mTau administered mice for 2 months. **B** Positive scotopic threshold response (pSTR) traces from control, glaucoma, glaucoma + AAV-GFP, and glaucoma + AAV-mTau overexpression mice retinas, as indicated and **C** their pSTR amplitudes quantification indicated a significant decline of the pSTR in the glaucoma AAV9-tau overexpression animals. (n = 10, ***P < 0.001 ****P < 0.0001) **D** Western blot analysis of Tau, pTau^S199/202^ and pTau^S404^ levels in control and glaucomatous retina. β-actin was used as a loading control **E** Fold change of Tau, pTau^S199/202^ and pTau.^S404^ showed a significant increase in retinas in high IOP conditions (***P < 0.001, n = 3 each group, t-test). **F** H and E staining of retinal sections of control, glaucoma, glaucoma + AAV9-GFP, and glaucoma + AAV9-mTau mice (representative images, arrows indicate the changes in cell densities, Scale bar, 50 μm). **G** Quantification of the cell density in GCL indicated a significant decrease in the number of cells in glaucoma and AAV-tau overexpressed retinas compared to the controls (n = 5, ****P < 0.0001, One-way ANOVA analysis with Tukey’s multiple comparison test)

The role of Tau protein modulation was further investigated in experimental model of glaucoma, where we used AAV9 therapy to specifically upregulate Tau expression in the RGCs. Mice eyes subjected to intracameral microbead injections showed a comparable increase in IOP across different mice groups (control, GFP and Tau overexpressing animals) (Fig. 3A). The inner retinal function was assessed using pSTR recordings (Fig. 3B). The results indicated that while glaucoma animals had a reduced pSTR amplitude (reduction of 44.28% ± 3.25, *P* < 0.001) compared to the control animals, the glaucomatous animals subjected to Tau upregulation demonstrated a further decline in pSTR amplitudes (reduction of 75.05% ± 3.28; *P* < 0.001) compared to the glaucoma eyes alone (Fig. 3C). No significant pSTR changes were detected in animals exposed to GFP alone expression. The whole retinal scotopic ERG measurements demonstrated no noticeable differences between the glaucoma eyes and glaucoma eyes subjected to either Tau overexpression or GFP expression (Fig. S3C). These results indicated that Tau over-expression-induced retinal electrophysiological changes were mainly localized to the inner retina.

We next evaluated mice retinal lysates by western blotting for Tau expression and pTau levels in glaucomatous conditions (Fig. 3D). A significantly higher expression of Tau, and increased pTau Ser^199/^Ser^202^ and pTau Ser^404^ levels (*P* < 0.001) were observed in high-IOP retinas compared to control mice (Fig. 3E). We further assessed the impact of Tau upregulation on the retinal laminar structure. Retinal sections were subjected to H and E staining and imaged microscopically (Fig. 3F). A significant decline in cell density in the GCL was observed both in the control glaucoma eyes (40.61% ± 7.75, *P* < 0.0001) as well as in the GFP sequence expressing glaucomatous eyes (41.64% ± 3.62, *P* < 0.0001). A further decline in GCL density was evident in the eyes that were subjected to Tau upregulation along with induction of experimental glaucoma (72.39% ± 5.5, *P* < 0.0001) (Fig. 3G).

### Tau silencing rescued inner retinal degenerative phenotype in experimental glaucoma model

**Fig. 4:**
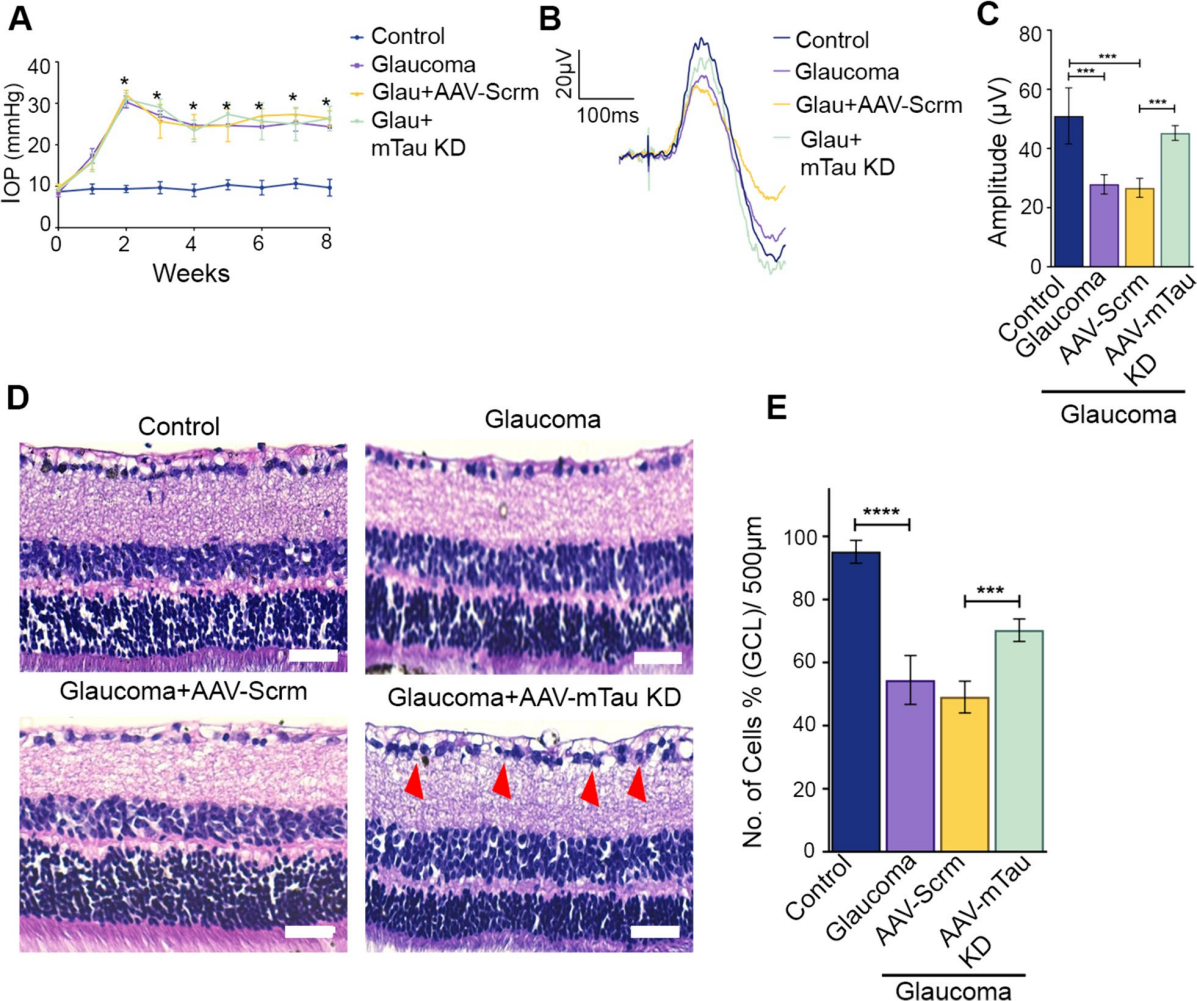
Silencing of Tau expression using AAV protects the inner retinal function and laminar structure in glaucomatous eyes. **A** Chronic elevation of IOP in C57BL/6 mice eyes was induced by intracameral microbead injections for 2 months. **B** Positive scotopic threshold response (pSTR) traces from control, glaucoma, glaucoma + scrambled and glaucoma + Tau KD mice retinas, as indicated. **C** Quantification of the pSTR amplitudes from the glaucoma AAV9-Tau KD mice eyes compared to glaucoma scrambled sequence expressing controls indicated significant protection against pSTR amplitudes loss in the glaucoma-AAV-Tau KD animals. (n = 10, ***P < 0.001, One-way ANOVA analysis with Tukey’s multiple comparison test). **D** H and E staining of retinal sections of the control, glaucoma, glaucoma + AAV9-scramble, and glaucoma + AAV9-tau KD mice eyes. (representative images, arrows indicate the changes in cell densities, Scale bar, 50 μm). **E** Quantification of the cell density in GCL indicated significant protection against cell loss in glaucoma AAV9-tau KD-subjected retinas compared to the microbead-injected control eyes. (n = 5, ***P < 0.001, ****P < 0.0001, One-way ANOVA analysis with Tukey’s multiple comparison test)

We further subjected glaucomatous animal retinas to Tau silencing using AAV9 constructs expressing shRNA targeted to Tau mRNA under the CAG2 hybrid promoter. The IOP increase upon intracameral microbead injections in different eye groups (control, scrambled and Tau shRNA expressing groups) was comparable (Fig. 4A). The inner retinal functional assessments indicated that Tau silencing imparted significant protection against the loss of pSTR amplitudes in glaucoma compared to the retinas subjected to AAV9 expressing scramble sequence (45.23 ± 2.50 v/s 26.72 ± 3.21 µV, *P* < 0.001) (Fig. 4B, C). No significant pSTR amplitude changes were detected in animals subjected to scramble sequence + glaucoma compared to the glaucoma only group (Fig. 4C). The whole retinal scotopic electroretinogram (ERG) measurements demonstrated no significant differences in a- and b-wave amplitudes between the glaucoma alone eyes and glaucoma eyes subjected to Tau silencing (Fig. S3D). These results indicated that Tau silencing-induced protection of retinal electrophysiological changes in glaucoma were mainly localized to the inner retina.

We further assessed the impact of AAV9 induced Tau silencing on the retinal laminar structure. Retinal sections were subjected to H and E staining, imaged and changes in cell density across GCL were quantified (Fig. 4D). A significant decline in the GCL cell density was observed in glaucomatous eyes (43.64% ± 9.84, *P* < 0.0001) and glaucoma + scramble sequence controls (46.01% ± 5.02, *P* < 0.0001). However, compared to the scramble sequence treated glaucoma animals, the loss of GCL density was reduced in the eyes that were subjected to Tau silencing along with microbead administration (24.86% ± 3.57 vs. 46.01% ± 5.02, *P* < 0.001) (Fig. 4E). These results suggest that Tau protein plays a crucial role in mediating inner retinal functional and structural degenerative changes in glaucoma conditions.

### Tubulin post-translational modification changes in response to Tau modulation

**Fig. 5:**
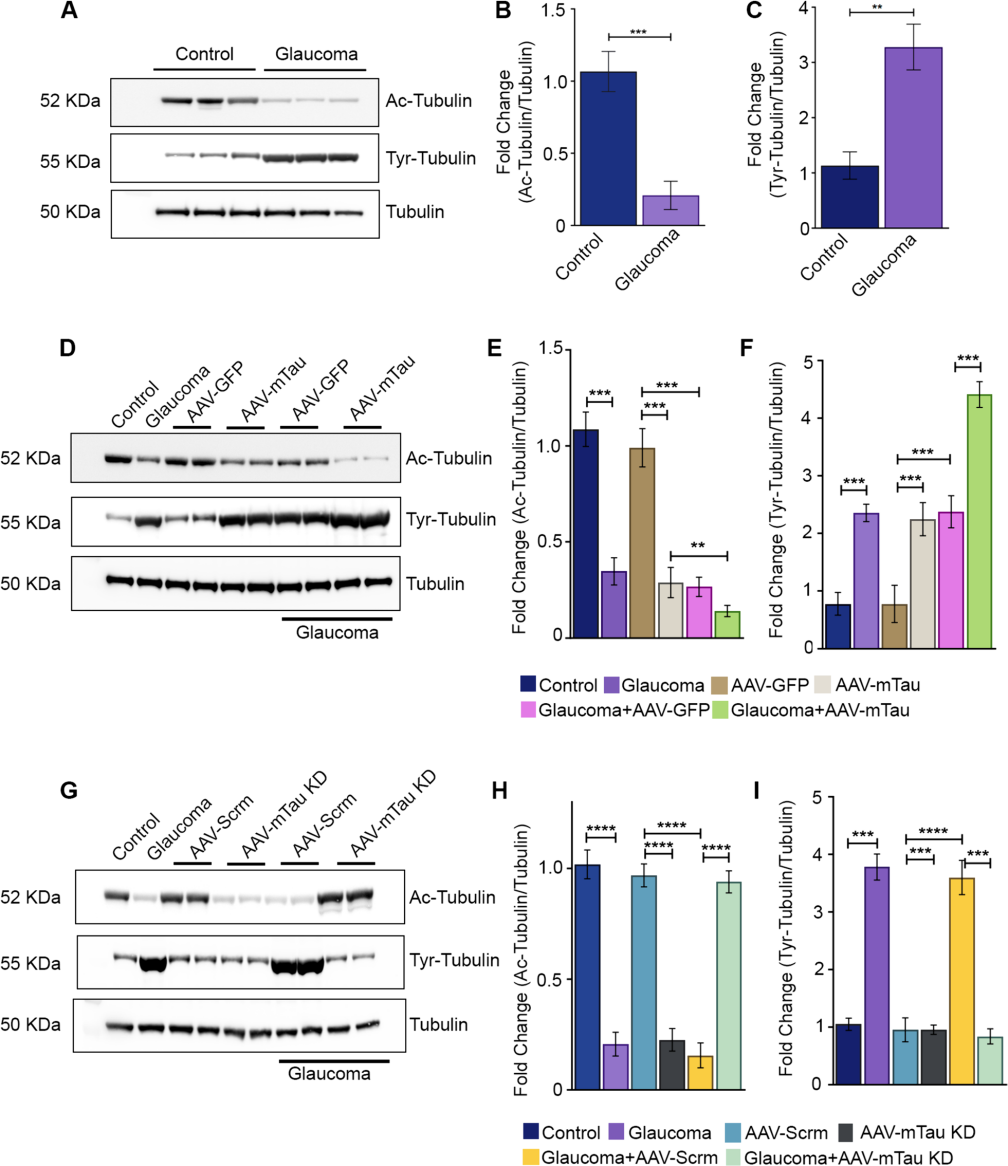
Western blotting (WB) analysis of tubulin and its post-translational modifications in AAV-mediated Tau modulated retinas. **A** WB of retinal lysates of the control and glaucomatous C57BL/6 mice eyes with Ac-tubulin (1:1000), Tyr-tubulin (1:1000), and total tubulin (1:1000) antibodies. **B** Quantification of relative intensities of the WB bands for Ac-tubulin and **C** Tyr-tubulin in retaliation to the total tubulin levels (n = 3, t-test, **P < 0.01, ***P < 0.001). **D** WB of retinal lysates of mice eyes transduced with AAV-GFP control and AAV-mTau construct (Tau overexpression) probed with Ac-tubulin, Tyr-tubulin, and total tubulin and their quantified **E** relative band intensities for Ac-tubulin and **F** Tyr-tubulin (**P < 0.01, ***P < 0.001, t-test, n = 3 per group, One-way ANOVA analysis with Tukey’s multiple comparison test,). **G** WB of retinal lysates from AAV-scrambled shRNA and AAV-mTau KD transduced mice probed with Ac-tubulin, Tyr-tubulin, and total tubulin antibodies. **H** Quantification of relative WB band intensities of Ac-tubulin and **I** Tyr-tubulin plotted to the total tubulin (n = 3) (***P < 0.001, ****P < 0.0001, t-test, n = 3 per group, One-way ANOVA analysis with Tukey’s multiple comparison test,)

The acetylation of tubulin is a hallmark feature in microtubule maturation and is considered a marker of microtubular stability. Western blotting analysis of retinal lysates revealed that acetylated form of tubulin (Ac-Tubulin) levels were significantly decreased in glaucoma conditions (*P* < 0.001) (Fig. 1A-B), and in the Tau overexpressing normal IOP retinas (*P* < 0.001) (Fig. 5D-E). Ac-Tubulin levels were further significantly reduced in the retinas of Tau overexpressing animals that were subjected to experimental glaucoma compared to the glaucoma eyes expressing GFP alone (*P* < 0.05) (Fig. 5E). In contrast, a significant increase in Ac-Tubulin levels was observed in the glaucoma eyes of animals subjected to Tau KD compared to glaucoma and AAV9 scrambled shRNA sequence administered glaucomatous eyes (*P* < 0.001) (Fig. 5G-H).

Protein tyrosination is another modification that occurs in tubulin and regulates microtubular interactions with microtubule-associated proteins and maintains neuronal survival and axonal integrity. Protein tyrosination also regulates microtubule dynamics and protein interaction with various subcellular structures. Increased levels of tyrosinated tubulin (Tyr–tubulin) have been shown to reflect degenerative changes and destabilisation of microtubular lattice [48]. In contrast to Ac–tubulin, which was significantly decreased in glaucoma eyes, Tyr-tubulin was observed to be significantly enhanced in the glaucoma retinas as determined by immunoblotting using specific antibodies (*P* < 0.01) (Fig. 5C). Our findings reveal a substantial elevation in Tyr-tubulin levels in animals overexpressing Tau under both normal IOP conditions (*P* < 0.001) and in glaucomatous eyes expressing GFP alone (*P* < 0.001) (Fig. 5F). The greatest increase in Tyr–tubulin levels was observed in the glaucomatous eyes of animals subjected to Tau overexpression experimental paradigm (*P* < 0.001) (Fig. 5F). Conversely, animals undergoing Tau knockdown exhibited a notable reduction in Tyr-tubulin levels in glaucomatous retinas (*P* < 0.001) compared to mice exposed to glaucoma alone and those treated with glaucoma + AAV9 scramble sequence. Tau knockdown in healthy eyes did not exhibit any significant alterations compared to control eyes and scramble sequence-treated control eyes (Fig. 5I).

### Tau modulation induced ER stress response

**Fig. 6:**
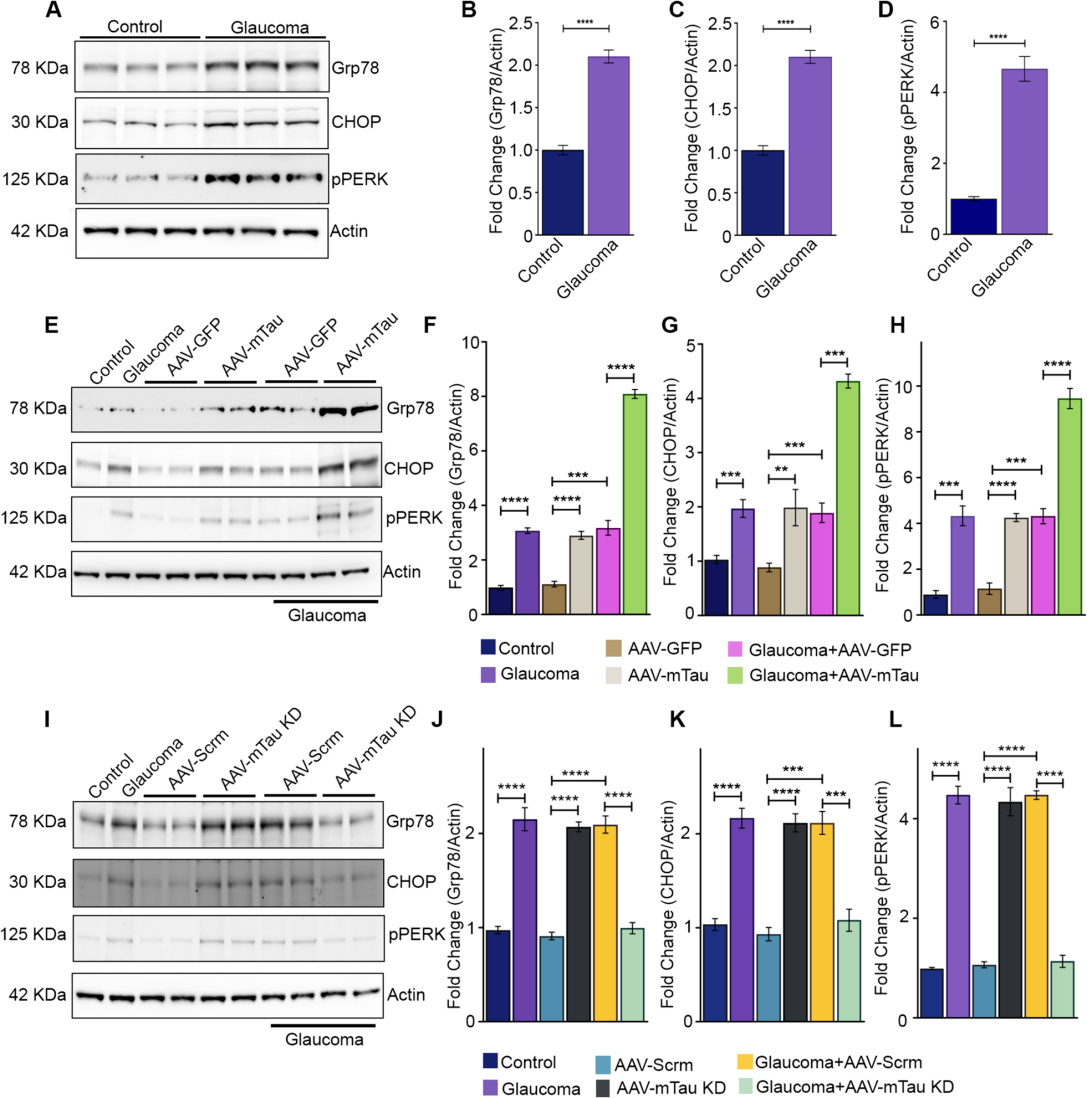
Western blot (WB) analysis of retinal endoplasmic reticulum (ER) stress markers changes in glaucoma and Tau modulation conditions. **A** WBs of control and glaucoma C57BL/6 mice retinal lysates probed with GRP78(1:1000), CHOP(1:1000), P-PERK(1:1000), and β-actin (1:5000) antibodies and **B-D** their respective relative band intensities quantified as the fold change using β-actin as loading control (n = 3 ****P < 0.0001, t-test). **E** WB of retinal lysates of the control, glaucoma, AAV-GFP, AAV-mTau (overexpression), AAV-GFP + glaucoma, and AAV-mTau + glaucoma mice were probed with GRP-78, CHOP, P-PERK, and actin antibodies, and **F-H** their respective relative band intensities quantified as the fold change using β-actin as loading control (n = 3, **P < 0.01, ***P < 0.001, ****P < 0.0001, One-way ANOVA analysis with Tukey’s multiple comparison test,). **I** WB of retinal lysates from control, glaucoma, AAV9-scramble, AAV-mTau KD, AAV9-scramble + glaucoma, AAV-mTau KD + glaucoma mice were probed with GRP 78, CHOP, P-PERK, and actin antibodies, and **(J-L)** their respective band intensities quantified as fold change using β-actin as a loading control (***P < 0.001, ****P < 0.0001, One-way ANOVA analysis with Tukey’s multiple comparison test, n = 3 per group)

Accumulating evidence suggests the involvement of tubulin post-translational modifications, such as acetylation and tyrosination, in cellular stress signalling responses [31]. Li et al. (2020), demonstrated that Ac-tubulin enhances cell survival during exposure to ER stress [27]. We examined changes in the expression of ER stress markers under normal and high intraocular pressure (IOP) glaucomatous conditions in response to AAV9 mediated Tau modulations. Immunoblotting analysis revealed a significant increase in GRP-78 (*P* < 0.0001), CHOP (*P* < 0.0001), and P-PERK (*P* < 0.0001) levels in glaucomatous eyes (Fig. 6A-D). Further, we observed significantly elevated GRP-78 (*P* < 0.0001), CHOP (*P* < 0.01) and P-PERK (*P* < 0.0001) levels in the retinas subjected to Tau overexpression (Fig. 6E-H) and Tau KD (*P* < 0.0001) under normal IOP conditions compared to the respective GFP and scrambled control groups respectively (Fig. 6I-L). Notably, retinal lysates from glaucomatous eyes subjected to Tau overexpression exhibited significantly enhanced levels of GRP-78 (*P* < 0.0001), CHOP (*P* < 0.001), and P-PERK (*P* < 0.0001) compared to glaucoma + GFP controls (Fig. 6F-H). In contrast, the ER marker GRP-78 (*P* < 0.0001), CHOP (*P* < 0.001) and P-PERK (*P* < 0.0001) levels were significantly reduced in glaucoma eyes subjected to Tau knockdown compared to either glaucoma controls or glaucoma mice subjected to AAV9-scrambled shRNA sequence control treatment (Fig. 6J-L).

### Tau silencing promotes retinal synaptophysin expression

**Fig. 7:**
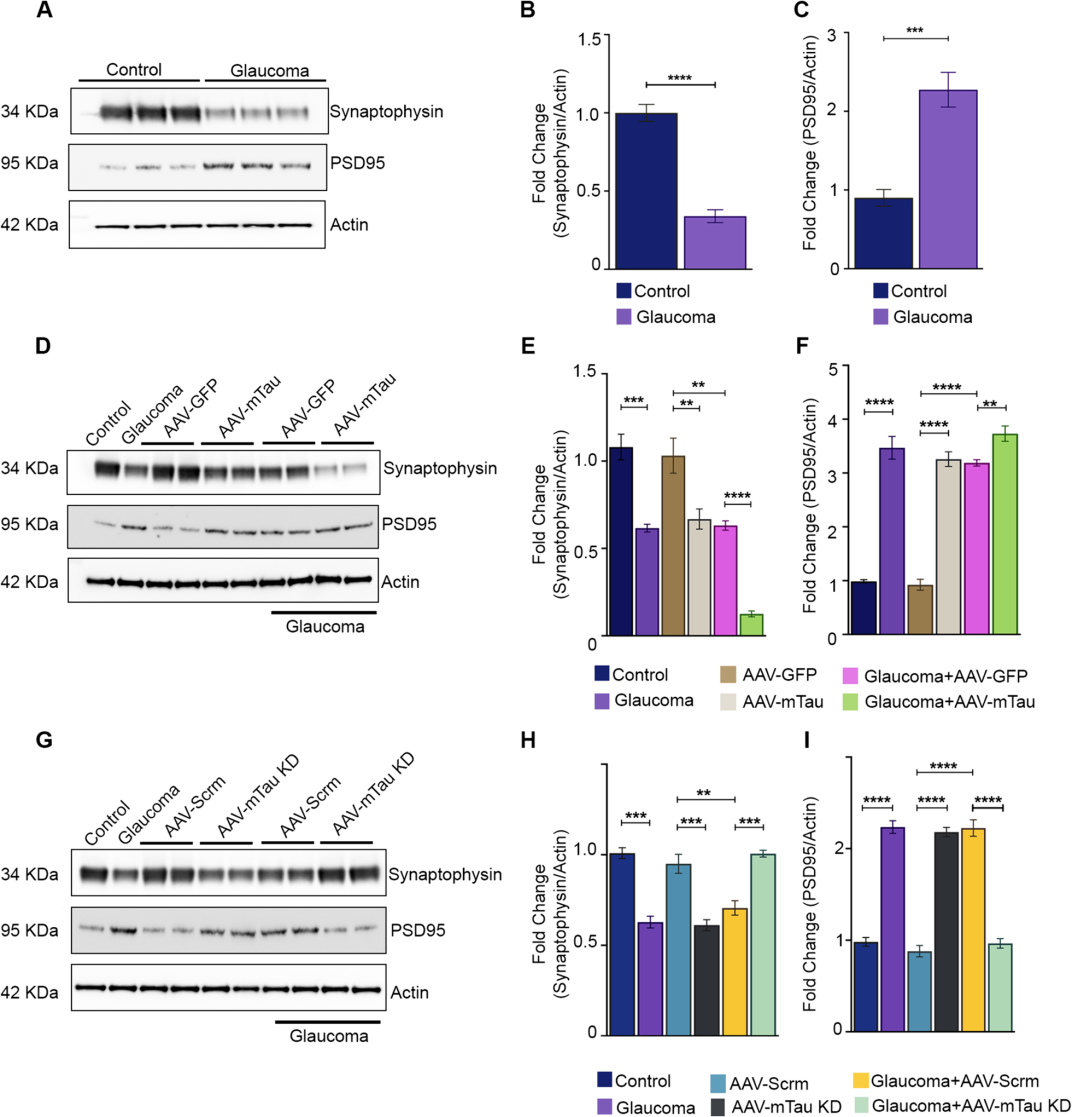
Western blotting (WB) analysis of synaptic signaling proteins changes in glaucoma and AAV-Tau modulations in the retina. **A** WB of glaucoma and control retinal lysates probed with anti-synaptophysin(1:20,000), anti-PSD95(1:1000), and β-actin (1:5000), antibodies. The band intensities were quantified using β-actin as a loading control and relative fold change plot for **B** Synaptophysin and **C** PSD95 (***P < 0.001, ****P < 0.0001 n = 3 each group, t-test). **D** WB of retinal lysates of AAV-GFP control and AAV-mTtau overexpression-subjected mice eyes probed with anti-synaptophysin and anti-PSD-95 antibodies in healthy and glaucoma conditions. The band intensities were quantified using β-actin as a loading control and relative fold change plotted for **E** synaptophysin and **F** PSD95 (**P < 0.01, ***P < 0.001, ****P < 0.0001, n = 3 each group, One-way ANOVA analysis with Tukey’s multiple comparison test). **G** WB of retinal lysates of AAV-Scramble control and AAV-Tau KD-subjected mice probed with anti-synaptophysin and anti-PSD-95 antibodies in normal and glaucoma conditions. The band intensities were quantified using β-actin as a loading control and relative fold change plotted for **H** Synaptophysin and **I** PSD95 (**P < 0.01, ***P < 0.001, ****P < 0.0001, n = 3 each group, One-way ANOVA analysis with Tukey’s multiple comparison test)

Since Tau protein expression is intricately associated with synaptic transmission integrity and axonal preservation, we next sought to investigate whether modulating Tau expression induces changes in the expression of synapse-associated proteins in the retina. To achieve this, we conducted immunoblot analysis on retinal lysates, focusing on the pre-synaptic protein synaptophysin, which regulates synaptic signalling and is abundantly expressed in synaptic vesicles. We also probed for PSD-95, a post-synaptic protein crucial for synaptic development and maintenance [8]. Our results demonstrated a significant reduction in synaptophysin expression under experimental glaucoma conditions (*P* < 0.0001) (Fig. 7A-C). Both Tau overexpression (*P* < 0.01) and Tau knockdown (*P* < 0.001) resulted in significantly decreased synaptophysin levels compared to controls with GFP alone and scrambled sequence administration, respectively, under normal intraocular pressure (IOP) conditions (Fig. 7D-I). Importantly, additional validation in the glaucoma animals superimposed with Tau overexpression paradigm, revealed a further reduction in synaptophysin expression compared to the control retinas (*P* < 0.0001) (Fig. 7E). Conversely, animals subjected to AAV9 Tau silencing in the experimental glaucoma paradigm exhibited protection against synaptophysin loss compared to controls expressing scrambled sequence (*P* < 0.001) (Fig. 7G, H). Furthermore, a significant increase in PSD-95 levels was observed under experimental glaucoma conditions (*P* < 0.001) (Fig. 7C). Both Tau overexpression and Tau knockdown also led to significantly increased PSD-95 levels (*P* < 0.0001) compared to controls with GFP alone and scrambled sequence administration, respectively, under normal IOP conditions (Fig. 7F, I). In glaucomatous animals subjected to Tau overexpression, a further increase in PSD-95 levels was evident (*P* < 0.01) (Fig. 7F). The Tau knockdown subjected glaucomatous cohort in contrast revealed significantly reduced levels of PSD-95 (*P* < 0.0001) compared to their respective scramble sequence treated controls (Fig. 7I).

### Tau therapy modulates Akt/Erk neuroprotective survival signalling pathway in the retina

**Fig. 8:**
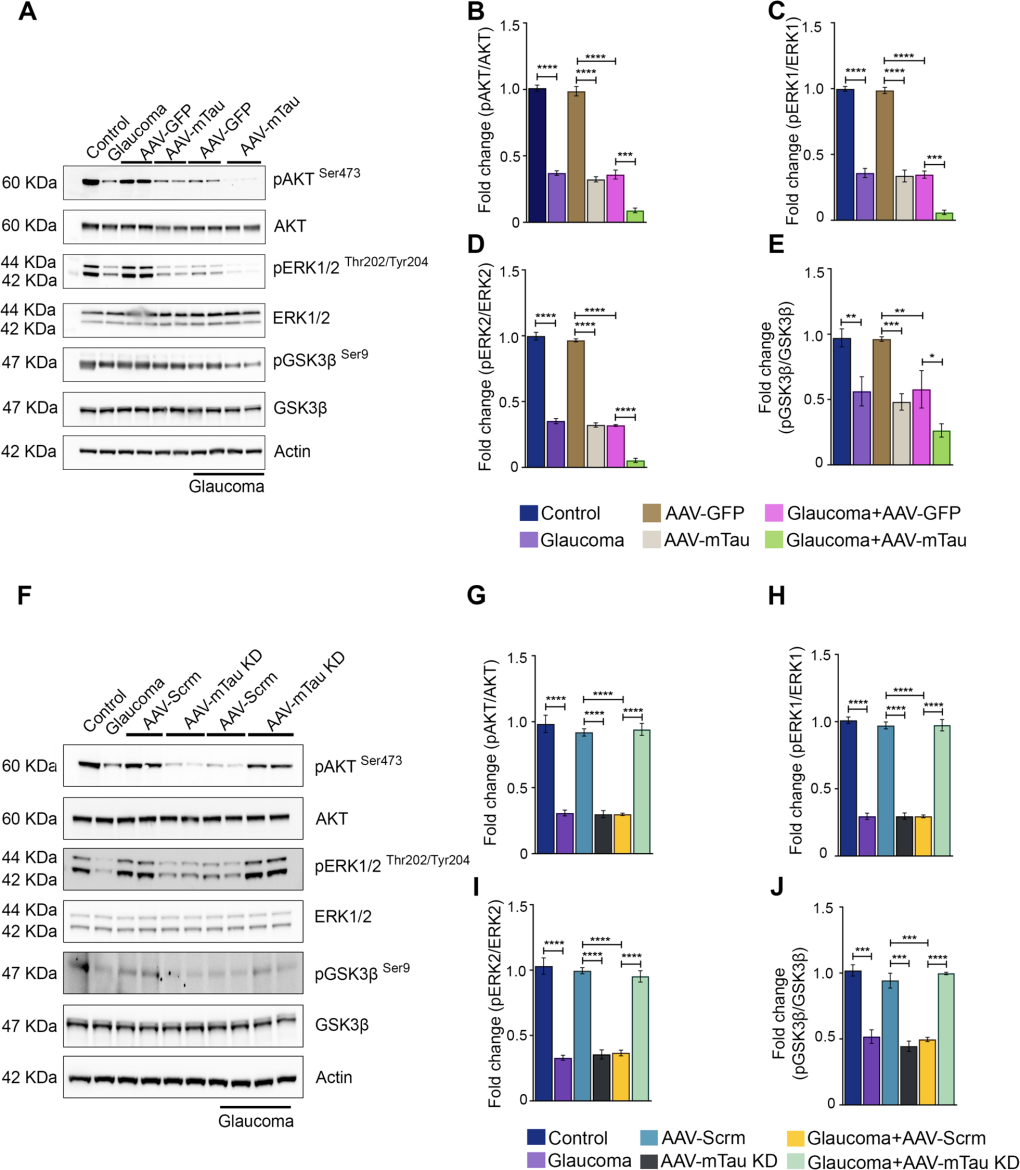
Western blot analysis of Akt, Erk and GSK3β signaling in the healthy and glaucoma retinas in response to AAV-Tau modulation. **A** Immunoblots of pAkt^(Ser473)^ (1:2000), Akt(1:1000), pErk^(Thr202/Tyr204)^ (1:2000), Erk(1:1000), pGSK3β^(Ser9)^(1:1000), and GSK3β(1:1000) in Tau overexpressing retinas in normal and glaucoma conditions compared to the respective control retinas and β-actin (1:5000), used as a loading control. **B** Quantification of pAkt^(Ser473)^ band intensities measures as relative fold changes to the total Akt expression. **C, D** Quantitative analysis of pErk1^(Thr202/Tyr204)^ and pErk2^(Thr202/Tyr 204)^ levels compared to the total Erk1 and Erk2. **E** Quantitative analysis of pGSK3β^(Ser9)^ band intensities and their relative fold changes compared to the GSK3β expression (*P < 0.05, **P < 0.01, ***P < 0.001, ****P < 0.0001, n = 3 each group, One-way ANOVA analysis with Tukey’s multiple comparison test). **F** Immunoblots of pAkt^(Ser473)^, Akt, pErk^(Thr202/Tyr204)^, Erk, pGSK3β^(Ser9)^, and GSK3β in AAV-mTau knockdown retinas in normal and glaucoma conditions compared to the respective control retinas and β-actin used as a loading control. **G** Quantitative analysis of pAkt^(Ser473)^ band intensities and its relative fold change to total Akt expression. **H, I** Quantitative analysis of pErk1^(Thr202/Tyr204)^ and pErk2^(Thr202/Tyr 204)^ compared to the total Erk1 and Erk2 levels. **J** Quantitative analysis of pGSK3β^(Ser9)^ band intensities to the total GSK3β expression (***P < 0.001, ****P < 0.0001, n = 3 each group, One-way ANOVA analysis with Tukey’s multiple comparison test)

Elevated Erk phosphorylation has been documented in response to Tau ablation in primary cortical neuronal cultures of mice [40]. Similarly, Akt/GSK3β pathway has been implicated in regulating Tau protein phosphorylation [9]. We sought to investigate the regulatory impact of Tau modulation on Erk and Akt/GSK3β phosphorylation in the retina in normal IOP and experimental glaucoma conditions. Immunoblotting analysis revealed decreased levels of pAkt Ser^473^, pErk and pGsk3β Ser^9^ in glaucomatous retinas compared to the control retinas (Fig. 8A). Furthermore, quantitative analysis unveiled a significant reduction in pAkt Ser^473^ (*P* < 0.0001), pErk Thr^202/^Tyr^204^ (*P* < 0.0001), and pGSK3β Ser^9^ (*P* < 0.001) reactivity in mice retinal lysates subjected to Tau overexpression compared to GFP expressing controls in normal IOP conditions. The loss of phosphorylation of these signalling molecules was further exacerbated when the Tau overexpression paradigm was combined with experimental glaucoma conditions pAkt Ser^473^ (*P* < 0.001), pErk Thr^202/^Tyr^204^ (*P* < 0.001), and pGSK3β Ser^9^ (*P* < 0.05) (Fig. 8B-E). We also assessed the impact of Tau silencing on the Erk and Akt/GSK3β signalling cascades in the mouse retina. Similar to the Tau overexpression studies, immunoblotting analysis revealed a significant decrease in pAkt Ser^273^ (*P* < 0.0001), pErk1/2 Thr^202/^Tyr^204^ (*p* < 0.0001), and pGSK3β Ser^9^ (*P* < 0.001) levels in the retinal lysates subjected to AAV mediated Tau silencing compared to AAV scrambled sequence controls under normal IOP conditions (Fig. 8F-J). Interestingly, the loss of phosphorylation of these signalling molecules was significantly rescued (*P* < 0.0001) under high IOP conditions when retinas were subjected to AAV9 Tau silencing, further reinforcing our previous findings that Tau protein silencing exerts a neuroprotective effect on the retina under experimental glaucoma conditions (Fig. 8G-J).

## Discussion

Tau, a protein abundantly expressed in the central nervous system (CNS), including the brain and retina, plays a crucial role in microtubular stabilization, axonal preservation, and the maintenance of synaptic functional integrity within axons [2, 30, 41]. Aberrant Tau hyperphosphorylation, coupled with increased formation of paired helical filaments (PHF) and neurofibrillary tangles, constitutes characteristic features of Alzheimer’s disease (AD) neuropathology [23, 42, 49]. Despite the recognized association of Tau accumulation and hyperphosphorylation with neurodegenerative changes, the causative role of Tau in inducing retinal neurodegenerative pathology in glaucoma remains unclear. The microtubule-associated protein Tau, enriched in axons, has previously been implicated in ganglion cell neurotoxicity in glaucoma [32, 35]. Increased Tau phosphorylation in retinas has been demonstrated in both human glaucoma subjects and rodent models of glaucoma [15, 18]. Recent studies reveal that exposure of eyes to elevated intraocular pressure (IOP) results in a rapid increase in retinal Tau, accompanied by an altered phosphorylation profile, mis-sorting, and the formation of Tau oligomers. Altered phosphorylation drives Tau accumulation and oligomerization, correlating with the presence of Tau oligomers in glaucomatous retinas [32, 35]. In glaucoma, Tau is predominantly reported in the somato-dendritic compartments of retinal ganglion cells (RGCs), suggesting Tau mis-localization and abnormalities in these regions [35]. Transgenic mice carrying the human P301S Tau mutant have previously demonstrated Tau aggregates in the retina, notably in RGCs, correlating with impaired anterograde and retrograde axonal transport, as well as increased susceptibility to excitotoxic damage [35]. These reports suggest an association between Tau aggregation and RGC dysfunction. However, It is essential to recognize that Tau transgenic mutant models may have inherent limitations, including development-related artifacts [19] and effects stemming from the loss or overexpression of Tau in different retinal cell types.

This study used AAV9 viral constructs to induce modulation of Tau expression in the mice retina. Tau modulation was achieved using both overexpression and silencing (Tau KD) approaches with GFP as a reporter sequence. AAV9 was delivered systemically via tail vein injection due to its ability to cross the blood brain barrier. Unlike intravitreal injections, which may result in uneven distribution and variable transduction efficiencies across different regions of the retina (central to periphery), systemic delivery of AAV9 promoted more consistent expression levels throughout the retina and minimized invasive effects of intravitreal injections on tissue including retinal injury, chronic inflammation, and risks of infections. The transduction efficiency and fold change in expression levels were established by monitoring GFP expression and evaluating changes in the Tau protein levels in the retina using western blotting and IF. The GFP expression was largely localized with NeuN, suggesting that Tau modulation was achieved primarily in the inner retinal layers. In addition to changes in the Tau protein levels in the retina in response to AAV9 transduction, we also observed significantly elevated levels of pTau Ser^199/202^ and pTau Ser^404^ in Tau overexpression subjected mice (Figs. 1 and S1). Conversely, there was a reduction of pTau Ser^199/202^ and Ser^404^ in Tau knockdown subjected retinal tissues in response to the silencing of Tau protein in these animals.

The pSTR measurements demonstrated significantly lower amplitudes in the Tau overexpressed and the Tau knocked down animals under normal IOP conditions (Fig. 2). As pSTR amplitudes reflect the electrophysiological function of the inner retina, results indicate that optimal Tau expression plays an important role in preserving the normal inner retinal function. The whole retinal scotopic ERG was not significantly altered, indicating that inner retinal function was predominantly affected (Fig. S3). Furthermore, H and E staining of the retinal sections provided evidence that functional losses corresponded with GCL loss in both Tau overexpression and Tau KD animals in normal IOP conditions (Fig. 2F).

In this study, we identified that corresponding to the Tau overexpression mice, animals subjected to AAV-mediated Tau knockdown demonstrated a decline in inner retinal function and thinning of the ganglion cell layer (Fig. 2). These results are corroborated by previous reports that Tau knockdown in the hippocampus can lead to learning and memory deficits and significantly lower dendritic spine density in pyramidal neurons [43]. Further, Tau deficiency has been shown to induce impaired fear conditioning and affect APP-mediated iron transport in mice [26]. As Tau knockdown leads to a significantly lower dendritic spine density in pyramidal neurons it can cause neuronal death and induce damage to neuronal functions. Increased Tau deposition has been observed in the retina of the mutated human P301S transgenic mice with evidence of retinal deficits [35]. Here, we determined whether the functional deficits in experimental glaucoma translated to anatomical deficits and studied the retinal laminar structural changes using H and E staining. A significant decline in the GCL density was evident under the experimental glaucoma conditions in Tau overexpression cohort. In the Tau knockdown animals, significant protection against the inner retinal functional and structural deficits was observed in experimental glaucoma conditions compared to the controls (Fig. 2). We further investigated changes in neuronal survival and stability by investigating the expression levels of tubulin, ER stress markers, and cell surviving pathway proteins in the retinal tissue.

Accumulating research underscores the pivotal role of Tau phosphorylation changes in preserving the protein's affinity for microtubules and determining the stability of the microtubular network [2, 21]. Tubulin proteins, crucial for intracellular transport, cell migration, mitosis, and cellular morphology maintenance, regulate these processes. Overexpression of h-Tau in mice and drosophila has been shown to enhance neuronal death and Tau filament detachment from the microtubules [2, 10, 49]. Tubulin post-translational modifications, including acetylation and tyrosination, serve as vital indicators of microtubular stability and maturation, synchronizing neurite outgrowth, development, and regulating neuronal morphology [48]. Acetylated tubulin is linked to stable and mature microtubular networks, crucial for maintaining neuronal stability. In neurodegenerative conditions, tubulin acetylation is negatively affected [13, 17, 27, 48]. Our observations revealed significantly reduced tubulin acetylation and increased tubulin tyrosination in glaucomatous retinas. AAV-Tau overexpression negatively impacted tubulin acetylation but enhanced tubulin tyrosination. These effects were exacerbated with Tau overexpression in glaucomatous conditions (Fig. 5). Conversely, AAV-Tau KD retinas exhibited decreased Tyr-tubulin levels in normal and high IOP conditions. Interestingly, tubulin acetylation levels were negatively affected in AAV-Tau KD under normal IOP conditions. However, Tau KD provided protection against this deficit in experimental glaucoma, where a higher tubulin acetylation level was evident, emphasizing Tau's role in mediating tubulin post-translational regulation (Fig. 5). Total tubulin levels remained unchanged in glaucoma and with Tau expression modulation. Tau hyperphosphorylation promotes pro-apoptotic pathways and cell senescence in AD [44]. Tau phosphorylation has also been shown to alter in various stress conditions, excitotoxicity and upon inhibition of lysosomal function in cortical neurons [29]. Elevated phosphorylated Tau levels were observed in glaucoma and Tau-overexpressing animal retinas under normal IOP, corresponding with increased p-PERK, Grp78 (Bip), and CHOP ER stress markers in both glaucoma and Tau-modulated conditions (Fig. 6). A significant exacerbation of ER stress marker expression was evident in the glaucoma animals subjected to Tau overexpression paradigm. Tau's role in modulating ER stress activation in glaucoma was further established in Tau KD studies, showing a significant decline in p-PERK, Bip, and CHOP protein expression compared to glaucoma eyes (Fig. 6).

There is significant evidence of synaptic dysfunction in animal models of Tauopathy [18, 44]. Evidence of synaptic loss has been shown in the hippocampus of P301S human Tau overexpressing transgenic mice even before NFT formation [46]. These transgenic animals demonstrated early synaptic loss with a significant decline in synaptophysin levels in the hippocampus. Synaptophysin is an integral membrane protein involved in presynaptic vesicle function in neurons and has been shown to be negatively impacted in various regions of the CNS in AD pathology [3]. This study identified synaptophysin downregulation in the retina in glaucoma conditions. A similar decline in synaptophysin was also observed in the AAV-Tau overexpression and Tau KD animals under normal IOP conditions (Fig. 7). Synaptophysin expression loss declined further when glaucomatous animals were subjected to AAV-Tau expression. However, induction of Tau KD in high IOP conditions protected against the loss of synaptophysin expression in the retina suggesting a regulatory effect of Tau on the presynaptic protein synaptophysin in the retina. Moreover, in this study we identified significantly increased levels of PSD-95 expression in glaucoma retinal lysates and in both Tau OV and KD conditions under normal IOP (Fig. 7). The PSD-95 levels were also elevated in the Tau overexpression eyes superimposed with the experimental glaucoma conditions. In the Tau silenced experimental glaucoma cohort significantly decreased PSD-95 levels were evident compared to the experimental glaucoma cohort (Fig. 7). These results suggest that both pre- and post-synaptic axonal biochemical milieu are affected in the retina in glaucoma and in response to Tau expression alterations suggesting signal transduction impairment in the retinal neurons in glaucoma that is mediated through the Tau protein.

The regulation of Tau interactions with microtubules involves a complex interplay of kinases and phosphatases, including GSK3β, Erk, and Akt. Akt signalling plays a crucial role in governing glucose metabolism, apoptosis, cell migration, and providing neuroprotection in a PI-3 kinase-dependent manner [38]. Activated Akt inhibits downstream pro-apoptotic pathways and caspase activation as well as the inhibition of pro-apoptotic factors such as Bcl-2-associated death domain proteins (BAD) [39]. Akt-induced Tau phosphorylation results in reduced binding of Tau with microtubular assemblies [37]. In this study, a substantial decrease in pAkt Ser473 levels in the retina was identified under glaucoma and Tau overexpression conditions (Fig. 8). Furthermore, when glaucomatous animals underwent AAV-Tau overexpression, pAkt Ser473 levels declined further, indicating the involvement of Tau expression changes in regulating pAktSer473 levels in the retina. Complementary results from AAV-Tau knockdown experiments demonstrated a significant decline in pAkt Ser^473^ levels under normal IOP conditions, emphasizing the importance of optimal Tau expression for maintaining retinal biochemical homeostasis. Notably, Tau knockdown in glaucomatous conditions provided significant protection against the loss in pAkt Ser473 levels, suggesting the usefulness of Tau silencing therapy as a protective strategy in glaucoma conditions. Akt activation exerts regulatory effects on GSK3β signalling, a factor implicated in mediating apoptotic pathways, microtubular dynamics, cell differentiation, and proliferation networks [24]. In glaucoma retinas, as well as under AAV-Tau overexpression and AAV-Tau knockdown in normal IOP conditions, a significant reduction in pGSK3β Ser9 levels was observed. This reduction was more pronounced in glaucomatous retinas subjected to Tau overexpression, indicating an additive effect of Tau overexpression on glaucoma pathology. In contrast, Tau silencing significantly enhanced pGSK3β Ser9 levels in glaucoma, reinforcing the neuropathological involvement of Tau in glaucoma conditions (Fig. 8). Beyond PI3K-Akt signaling, Erk plays a role in regulating axonal growth, neuroprotection, and the survival of retinal ganglion cells (RGCs) [5, 7]. Erk activation has also been implicated in the regulation of apoptosis by blocking caspase activation [47]. We observed similar results where reduced pErk(Thr^202^/Tyr^204^) levels were evident in glaucomatous retinas and in response to AAV-Tau overexpression. The pErk levels were further reduced when AAV-Tau overexpression conditions were superimposed with high IOP-induced glaucoma effects. Conversely, although AAV-Tau KD, negatively affected the pErk levels, Tau silencing in glaucoma conditions rescued the loss of pErk(Thr^202^/Tyr^204^) in the retina, thus further establishing the protective effects of Tau silencing in glaucoma neuropathology. The detrimental effects of Tau silencing in normal physiological conditions suggest that optimal protein levels are essential to preserve retinal integrity. This is expected to pose a practical challenge when modulating the Tau levels via gene therapy approaches.

While this study sheds light on the modulation of Tau expression in mouse retinas and offers valuable insights into the pathophysiology of glaucoma, it has certain limitations. Human glaucoma is characterized by non-Mendelian inheritance and involves a complex interplay of intraocular pressure, vascular, genetic and lifestyle factors. In contrast, rodent models mimic only certain aspects of the glaucomatous pathology that may not fully recapitulate the complex manifestations observed in human patients. Moreover, variances may arise from anatomical dissimilarities, genetic distinctions, and species-specific differences in physiological responses. Nevertheless, this study provides compelling evidence for the role of tau in glaucoma conditions and offers valuable insights into mechanistic aspects of disease pathology. In summary, this study underscores the essential role of optimal Tau expression in maintaining the retinal structural and functional homeostasis and preserving the retinal biochemical milieu. Since Tau levels are affected in glaucoma, AAV-Tau silencing therapy could be explored as a strategy to mitigate the detrimental effects of Tau accumulation and provide neuroprotection.

### Supplementary Information

Supplementary Material 1.

## Data Availability

The authors declare that all the relevant data, associated protocols, and materials supporting the findings of this study are present in the paper.
