# Supplementary material for: Tau modulation through AAV9 therapy augments Akt/Erk survival signalling in glaucoma mitigating the retinal degenerative phenotype
Source: Acta Neuropathol Commun. 2024 Jun 7;12:89. doi: 10.1186/s40478-024-01804-0 (PMC11158005; doi:10.1186/s40478-024-01804-0)
Supplement: Supplementary file 1 — Supplementary Material 1. [file 40478_2024_1804_MOESM1_ESM.docx]

**Supplementary Figures for**

**Tau Modulation through AAV9 Therapy Augments Akt/Erk Survival Signalling in Glaucoma mitigating the Retinal Degenerative Phenotype**

**This file includes:**

Supplemental Figures S1-S3


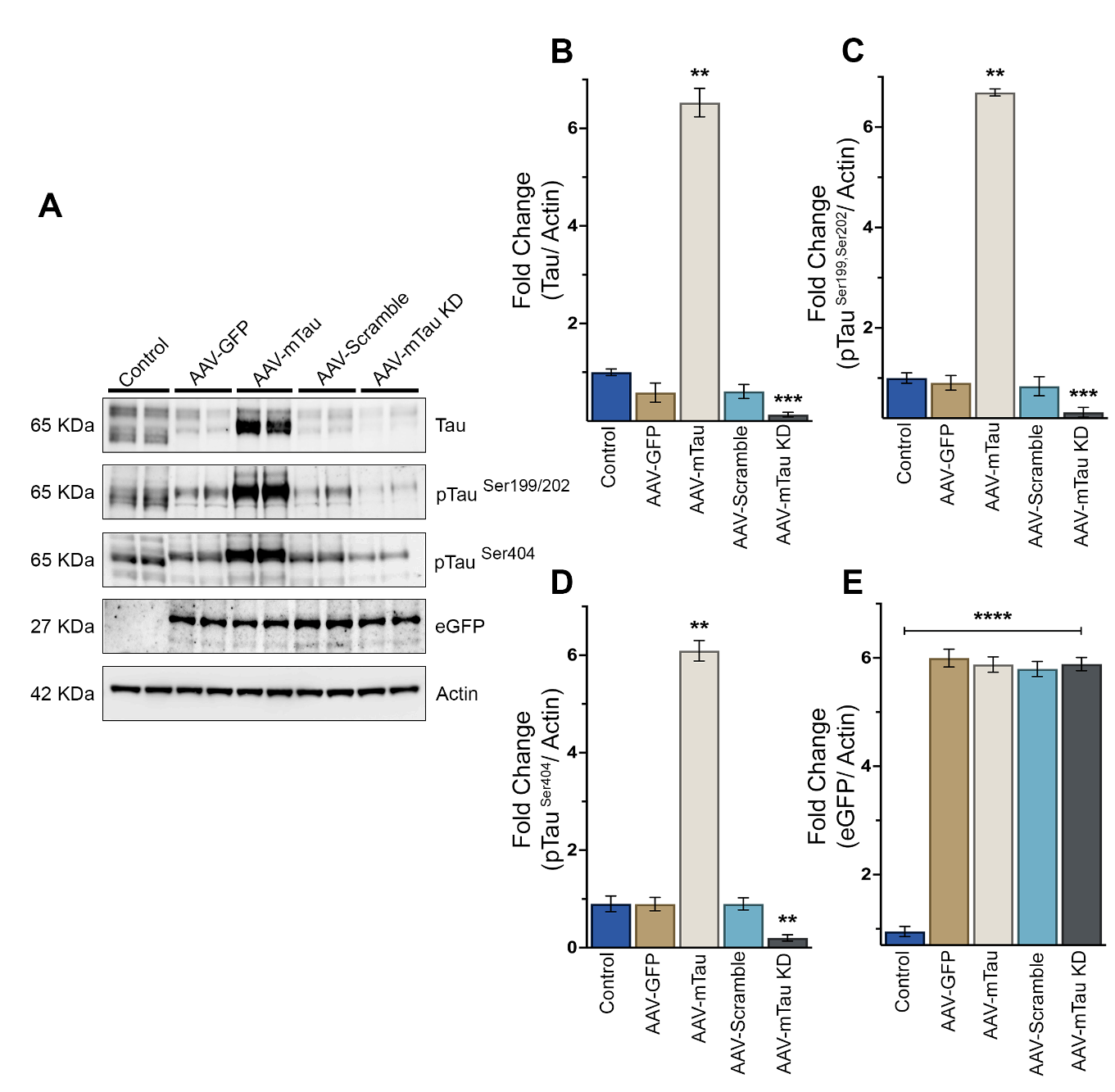


Supp Figure S1. Western blot analysis of Tau, pTau, and GFP expression levels from AAV-mTau-overexpression and AAV-mTau-knockdown (KD) subjected mice retinas compared to the controls. (A) Immunoblots of retinal lysates probed with Tau, pTau^Ser199/Ser202^(1:1000), pTau^Ser404^(1:1000), GFP(1:1000), and β-actin (1:5000) antibodies and (B-D) Quantitative analysis of relative band intensities of Tau, pTau^Ser199/Ser202^ and pTau^Ser404^ plotted using β-actin as a loading control. (E) Expression of GFP levels was quantified in the WB across all the AAV-transduced retinas and compared with the controls. (n = 3 each, p**<0.0001, p***<0.0001, p****<0.0001, One-way ANOVA analysis with Tukey’s multiple comparison test).


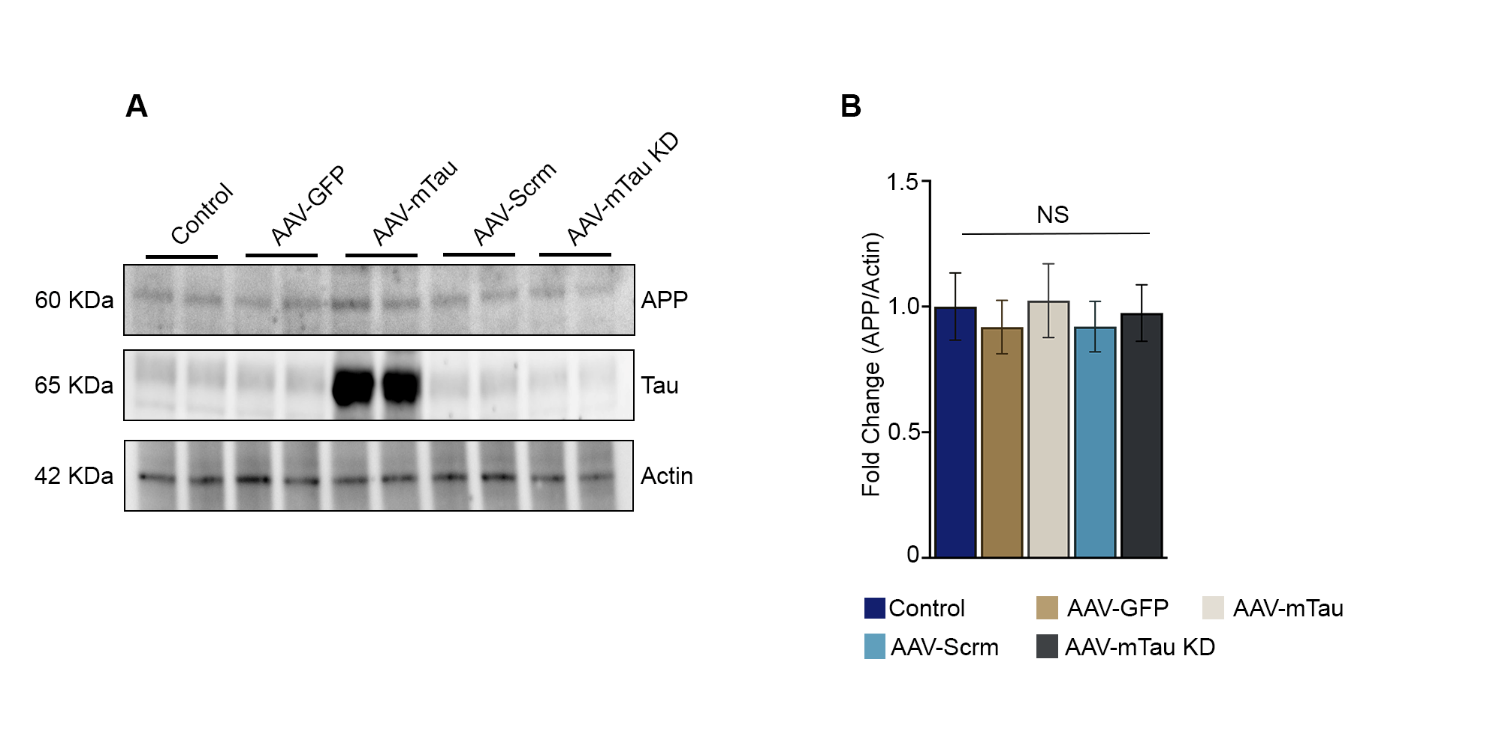


Supp Figure S2. Western blot analysis of APP and Tau expression levels in AAV subjected mice retinas compared to the controls. (A) Immunoblots of retinal lysates probed with Tau (1:1000), APP (1:1000), and β-actin (1:5000) antibodies and (B) Quantitative analysis of relative band intensities of APP plotted using β-actin as a loading control (n = 3 each, NS).


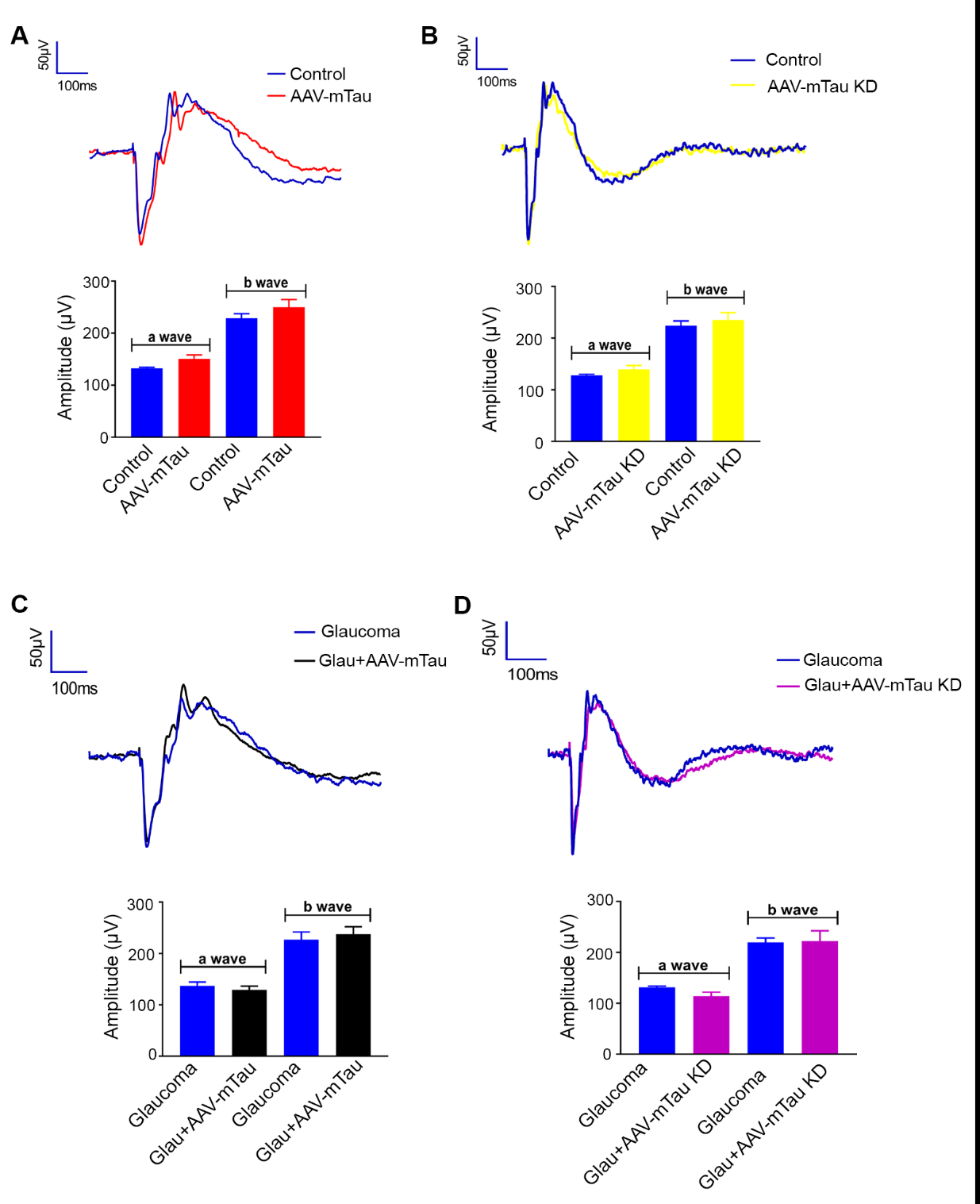
Supp Figure S3. Electroretinogram recordings of control and AAV-Tau modulated mice eyes in normal and glaucoma conditions. (A) Average ERG traces of control and AAV-mTau overexpression mice eyes in normal IOP conditions. Data analyses of ERG a (blue)- and b (red)-wave amplitudes revealed no significant differences between the amplitudes of control and AAV-mTau overexpressing mice (B) Average ERG traces of control and AAV-mTau KD mice eyes in normal IOP conditions. Data analyses of ERG a (blue)- and b (yellow)-wave amplitudes revealed no significant differences between the amplitudes of control and AAV-mTau KD mice (C). Average ERG traces of glaucoma and glaucoma+AAV-mTau overexpression in high IOP conditions. Data analyses of ERG a (blue)- and b (black)-wave amplitudes revealed no significant differences between the amplitudes of glaucoma and glaucoma+AAV-mTau overexpressing mice D. Average ERG traces of glaucoma and glaucoma+AAV-mTau KD in high IOP conditions. Data analyses of ERG a (blue)- and b (magenta)-wave amplitudes revealed no significant differences between the amplitudes of glaucoma and glaucoma+AAV-mTau KD mice (n=10 mice in each group).
